# EEG Artifact Removal in TMS Studies of Cortical Speech Areas

**DOI:** 10.1007/s10548-019-00724-w

**Published:** 2019-07-09

**Authors:** Karita S.-T. Salo, Tuomas P. Mutanen, Selja M. I. Vaalto, Risto J. Ilmoniemi

**Affiliations:** 1 Department of Neuroscience and Biomedical Engineering, Aalto University School of Science, P.O. Box 12200, 00076 AALTO Espoo, Finland; 2 BioMag Laboratory, HUS Medical Imaging Center, Helsinki University Hospital and University of Helsinki, P.O. Box 340, 00029 HUS Helsinki, Finland; 3 Centre for Cognitive Neuroimaging, Institute of Neuroscience and Psychology, University of Glasgow, Glasgow, G12 8QB UK; 4 Department of Clinical Neurophysiology, HUS Medical Imaging Center, Helsinki University Hospital and University of Helsinki, P.O. Box 340, 00029 HUS Helsinki, Finland

**Keywords:** Transcranial magnetic stimulation, Electroencephalography, Signal-space projection, Source-informed reconstruction, Broca’s area, Wernicke’s area

## Abstract

The combination of transcranial magnetic stimulation (TMS) and electroencephalography (EEG) is commonly applied for studying the effective connectivity of neuronal circuits. The stimulation excites neurons, and the resulting TMS-evoked potentials (TEPs) are recorded with EEG. A serious obstacle in this method is the generation of large muscle artifacts from scalp muscles, especially when frontolateral and temporoparietal, such as speech, areas are stimulated. Here, TMS–EEG data were processed with the signal-space projection and source-informed reconstruction (SSP–SIR) artifact-removal methods to suppress these artifacts. SSP–SIR suppressed muscle artifacts according to the difference in frequency contents of neuronal signals and muscle activity. The effectiveness of SSP–SIR in rejecting muscle artifacts and the degree of excessive attenuation of brain EEG signals were investigated by comparing the processed versions of the recorded TMS–EEG data with simulated data. The calculated individual lead-field matrix describing how the brain signals spread on the cortex were used as simulated data. We conclude that SSP–SIR was effective in suppressing artifacts also when frontolateral and temporoparietal cortical sites were stimulated, but it may have suppressed also the brain signals near the stimulation site. Effective connectivity originating from the speech-related areas may be studied even when speech areas are stimulated at least on the contralateral hemisphere where the signals were not suppressed that much.

## Introduction

Transcranial magnetic stimulation (TMS) excites neurons noninvasively below the stimulation coil (Barker et al. 1985); combined with electroencephalography (EEG), TMS can be applied to study effective connectivity, i.e., causal connections between different cortical areas (Ilmoniemi et al. 1997; Komssi et al. 2002; Massimini et al. 2005). However, the strong TMS pulse activates also scalp muscles, which gives rise to large artifacts in the EEG signal (Ilmoniemi and Kičić 2010; Mutanen et al. 2013). Large muscles such as those located in the lateral sides of the head generate EEG artifacts that can be up to 1000 times the size of the neuronal EEG signal and can last tens of milliseconds (Rogasch et al. 2013; Mutanen et al. 2013). These muscle artifacts can make it hard or impossible to interpret the TMS-evoked potentials (TEPs) recorded with EEG (Nikulin et al. 2003; Rosanova et al. 2009; Cona et al. 2011; Farzan et al. 2013), especially when frontolateral and temporoparietal cortical areas are stimulated. This has made it difficult to use TMS–EEG for studying speech areas (Wernicke’s and Broca’s areas) and their connectivity. However, the reliable estimation of effective connectivity parameters (Salo et al. 2018) within the language network could improve our understanding of the interplay and functions of the various speech-related cortical areas. Hence, this information could be directly used in the planning of therapeutic TMS protocols to treat language impairments, for example, those caused by stroke or other pathophysiologies (Hamilton et al. 2011; Carreiras et al. 2012; Thiel et al. 2013; Heikkinen et al. 2019).

One approach to suppress muscle artifacts is signal-space projection (SSP) (Mäki and Ilmoniemi 2011; Hernandez-Pavon et al. 2012; Mutanen et al. 2016); this approach is based on estimating the signal subspace containing muscle artifacts to form a linear operator that would remove these artifacts from the measured data. SSP introduces some attenuation in the cortical EEG signals of interest (Mäki and Ilmoniemi 2011), making the visual interpretation of the remaining EEG more difficult. Recently, an additional source-informed-reconstruction (SIR) step was introduced to minimize the SSP-caused attenuation in the displayed EEG signals (Mutanen et al. 2016).

We evaluated the ability of SSP–SIR to suppress muscle artifacts when cortical speech areas are stimulated. We also investigated how EEG signals arising from the brain are affected as a side effect of SSP–SIR. The results were compared with those of motor-area stimulation, in which SSP–SIR has proven to be effective (Mutanen et al. 2016). We stimulated three sites of the right hemisphere and compared the resulting TMS-evoked potentials (TEPs) before and after SSP–SIR. The decision of stimulating the right hemisphere was based on the fact that stroke-related aphasia treatments are usually delivered to the right hemisphere to balance the interhemispheric activity (Turkeltaub 2015). Thus, the connectivity between the right and left hemispheres should be elucidated, for targeting repetitive TMS (rTMS) optimally in therapeutic interventions in the future.

## Materials and Methods

### Subjects

Three right-handed volunteers (S1, female, 25 years old; S2, male, 27; S3, male, 30) participated in the experiment, which had been accepted by the Ethics Committee of Helsinki University Hospital and was compliant with the Declaration of Helsinki. The research procedures were explained to the subjects, who gave written informed consent before the experiment.

### EEG and TMS

TEPs were recorded with a 60-channel EEG cap and a TMS-compatible eXimia EEG device (Nexstim Plc, Helsinki, Finland). The impedances of the electrode contacts were prepared to be < 15 kΩ. The reference electrode was attached to the forehead, the ground electrode to the right zygomatic bone, and the electrooculography electrodes just above the right eyebrow and on the left side of the left eye. Individual MRIs and navigated brain stimulation (NBS 4.3, Nexstim) were applied to track the location of the stimulation coil with respect to the head. Motor evoked potentials (MEPs) of the left abductor pollicis brevis (APB) muscle were recorded with a Nexstim electromyography (EMG) system.

A Nexstim TMS stimulator with a sample-and-hold circuit (Virtanen et al. 1999), to prevent TMS-induced artifact saturation of the amplifier, and a figure-of-eight coil were used to deliver sequences of biphasic TMS pulses targeted to the right hemisphere with NBS, while the EEG signals were recorded with a passband of 0.1–350 Hz and sampled at the rate of 1450 Hz. A piece of 1-cm-thick foam plastic was placed between the scalp and the coil to diminish the sensory stimulation of the scalp and auditory evoked potentials (Gordon et al. 2018; Conde et al. 2019). Auditory evoked potentials were minimized by hearing protection and by masking the sound of the TMS coil with white noise via headphones (Nikouline et al. 1999). The representation area of the left APB in the right primary motor cortex (M1) was mapped first, and the resting motor threshold (rMT) was determined as the smallest stimulator intensity that in ten trials produced at least five MEPs with peak-to-peak amplitudes of at least 50 µV (Rothwell et al. 1999) in APB. The frontolateral and temporoparietal stimulation sites in the right hemisphere corresponding to Broca’s (opercular inferior frontal gyrus, opIFG) and Wernicke’s (superior temporal gyrus, STG) areas in the left hemisphere were determined based on anatomical landmarks (see the section: “Stimulation sites”). Each subject received 150 stimuli to each target at random intervals varying between 3.0 and 3.5 s with a stimulation intensity that was equal to the corresponding E-field value of 90% of rMT. The relatively low stimulation intensity was chosen to minimize EEG contamination from muscle artifacts (Mutanen et al. 2013) and any motor activation feedback responses.

### Lead-Field Matrix, Simulated Data, and Stimulation Sites

The locations of the reference and EEG electrodes were digitized with NBS to enable the construction of the subject-specific lead-field matrix $\bf{L}$ (60 × 5124). ${\bf{L}}$ describes how brain sources generate potential differences between electrodes according to the knowledge on an individual level of the form and conductivity of each tissue type from the brain to the scalp and the locations of the electrodes. The column vectors of ${\bf{L}}$ describe the signal topographies of EEG-generating sources that would lie on the cortical gray–white-matter surface. In turn, the row vectors of ${\bf{L}}$ correspond to the cortical sensitivity profiles of EEG sensors. The pipeline for the construction of the anatomical models and the lead-field matrices is presented in detail in (Salo et al. 2018). Briefly, the methods introduced in (Fischl et al. 2002) and (Shattuck and Leahy 2002) were applied to segment anatomical MR images and those in (Stenroos and Sarvas 2012) to build a three-compartment forward model and to solve lead fields for cortically constrained sources. The topographies generated by cortical sources (post-synaptic currents) described with ${\bf{L}}$ were used as simulated data to show how EEG signals generated at different locations on the cortex are attenuated by SSP–SIR.

**Fig. 1:**
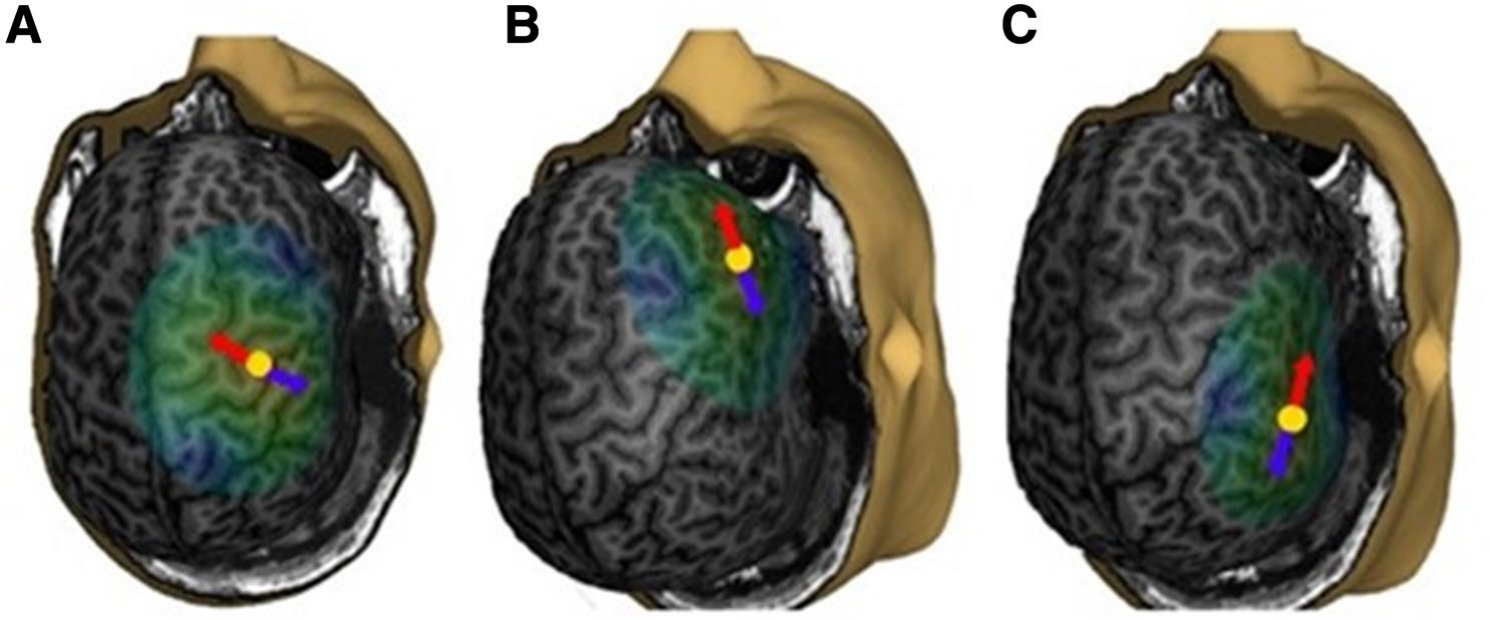
The stimulation targets of S3. **a** The right M1. **b** The right opIFG. **c** The right STG. The yellow dot indicates the location and the red arrow the direction of the activating E-field

The TMS target in the right M1 in the precentral gyrus that produced the highest MEPs in the left APB was chosen to be the M1 stimulation site. The activating E-field (and also the induced current) at the target site was oriented towards the precentral gyrus. The two other stimulation sites were chosen according to the individual anatomy. OpIFG in the inferior part of the left frontal lobe (in right-handed individuals) consists of pars opercularis (F3Op), pars triangularis (F3Tr), and pars orbitalis (F3Or) (Skipper et al. 2007; Keller et al. 2009). When stimulating the anatomically corresponding area in the right hemisphere, stimulation was targeted to the sulcus between pars opercularis and pars triangularis, the activating E-field being oriented anteriorly towards pars triangularis. The stimulation was targeted to the posterior end of STG (Dewitt and Rauschecker 2013) or the angular gyrus with the E-field towards the angular gyrus or STG. As the border between these gyri cannot be clearly separated, the exact anatomical definition of the stimulated area cannot be given. The stimulation targets are shown in Fig. 1.

### Data Processing

**Fig. 2:**
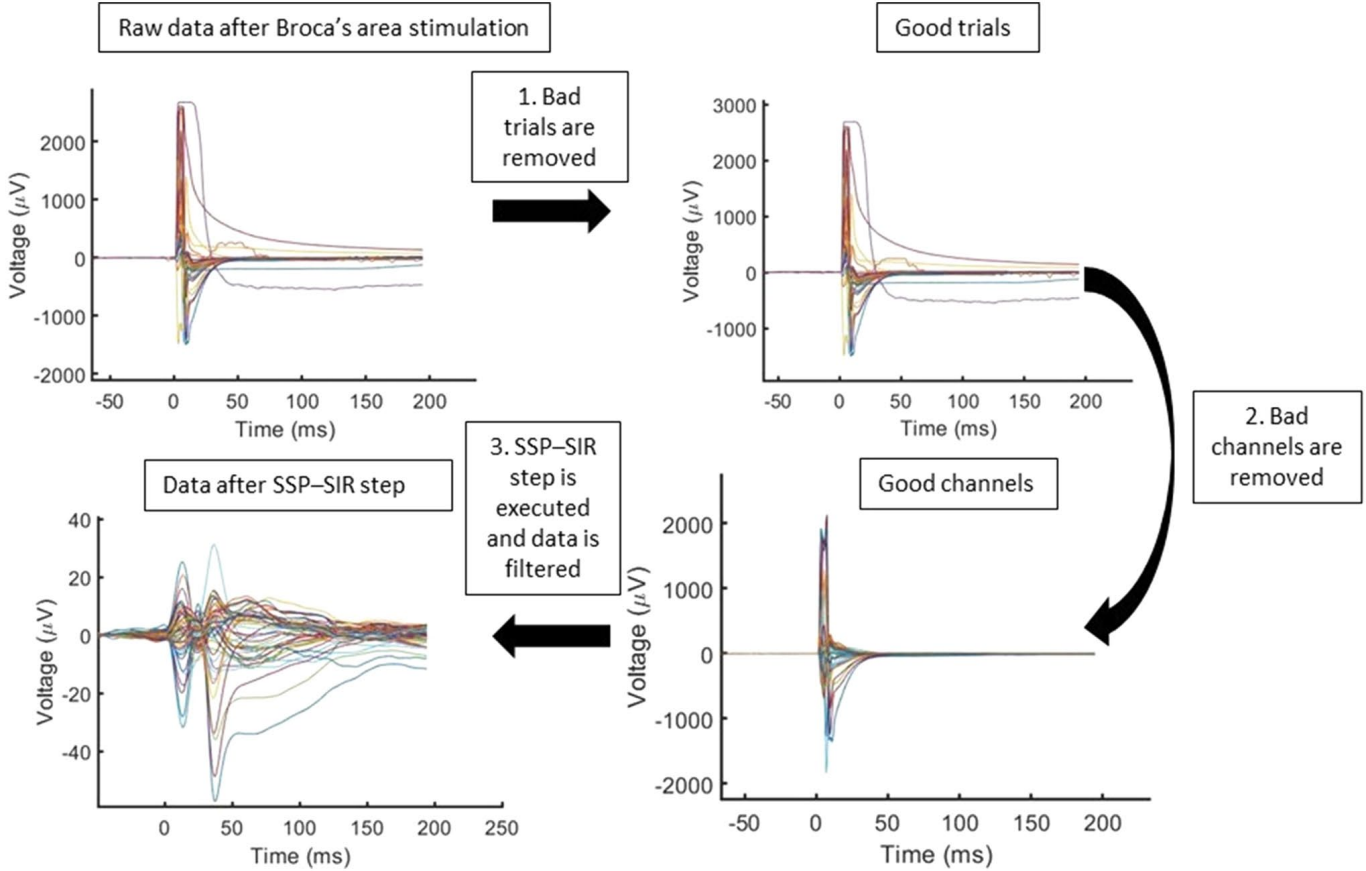
The artifact-removal steps and the effect of each of these steps on the butterfly plot for opIFG stimulation of S1

The data, processed offline with MATLAB R2018b (The Mathworks, Inc., Natick, MA), were segmented into trials from − 300 to 500 ms with respect to the TMS stimulus. Based on the visual evaluation, bad channels and bad trials with random artifacts, such as ocular artifacts (Ilmoniemi and Kičić 2010), were removed since these channels and trials brain signals might have been contaminated with the artifacts. The data were then averaged over the accepted trials and average-referenced to remove any errors affecting all the channels. Next, SSP–SIR (Mutanen et al. 2016) with a realistic head model was applied to suppress muscle artifacts by focusing the procedure to the time window with the largest artifacts using a SVD truncation level calculated by subtracting the number of removed channels and one dimension for average reference from 60 dimensions. Thus, the late muscle-artifact-free TEP components remained unaffected by SSP–SIR, and therefore, were not used here to assess the effects of SSP–SIR on the data. First, a projection matrix **P** was computed from the high-pass-filtered data assuming that only a negligible part of the EEG signals above 100 Hz is due to brain activity and the most of it is due muscle activity as described in (Mäki and Ilmoniemi 2011). The data **Y** and lead-field matrices **L** were multiplied by **P** to suppress the effect of muscle artifacts. The source estimates were then computed from the cleaned, artifact-free data, **PY**, using suppressed lead-field matrix, **PL**. The SSP–SIR step gives a correction matrix that can be used to execute the step by multiplying the data with it. These source estimates with the original lead-field matrix were used for the reconstruction of the evoked brain signals in the original EEG channels (Mutanen et al. 2016). The detailed description of this method can be found in (Mutanen et al. 2016). Finally, bandpass filtering with a zero-padded Butterworth filter was applied to the data at 2–80 Hz to only include frequencies with brain activity. The steps in artifact-removal have been shown in Fig. 2.

### Analysis

The recorded TMS–EEG data and the simulated EEG data were analyzed as described next. The first analysis was done to observe if the typical TEP components could be found after the artifact-removal. The second analysis was done to see how much SSP–SIR suppressed the brain signals and if there was no suppression, it could be concluded that the corresponding signals could be used for, e.g., source estimation analysis.

#### TMS–EEG Analysis

This analysis was performed to verify that SSP–SIR is capable of retaining the neuronal TEPs; global mean-field amplitudes (GMFA) (Lioumis et al. 2009) were calculated for each dataset as was done in (Salo et al. 2018) to see if the typical TEP components can be identified. Then, the location of the cortical source of the first recognized TEP component was estimated by finding the dipole (fitted dipole) that would best describe the earliest TEP component visible in the cleaned data. The distance between the fitted dipole and the stimulation site was determined. The topography of the first TEP component in each dataset was compared with the topography of the fitted dipole by calculating a goodness-of-fit value (GOF). GOF was determined as:

1 $${\text{GOF}} = 1 - \frac{{\mathop \sum \nolimits_{k} \left( {{\text{y}}_{{k}} - {\hat{\text{y}}}_{{k}} } \right)}}{{\mathop \sum \nolimits_{k} \left( {\text{y}}_{{k} } \right)^{2} }}^{2} ,$$

where *y*_*k*_ is the measured signal and $\hat{\text{y}}_{{k}}$ is the amplitude calculated from the fitted dipole in the *k*th channel.

#### Simulation Analysis

We calculated the correlation coefficient (CC) and relative difference (RD) between the simulated data and the same data processed with the SSP–SIR operator. The idea was to process the simulated data as if it had the same muscle artifacts as the measured TEPs to quantify the SSP–SIR-caused changes in the EEG signals of interest. The calculations were done as follows:

2 $${\text{CC}}_{i} = \frac{{\left( {{\mathbf{SSPSIR}} \times {\mathbf{L}}_{i} } \right)^{\text{T}} {\mathbf{L}}_{i} }}{{\left| {{\mathbf{SSPSIR}} \times {\mathbf{L}}_{i} } \right|\left| {{\mathbf{L}}_{i} } \right|}}$$

3 $${\text{RD}}_{i} = \frac{{\left| {{\mathbf{SSPSIR}} \times {\mathbf{L}}_{i} - {\mathbf{L}}_{i} } \right|}}{{\left| {{\mathbf{L}}_{i} } \right|}},$$

where ${\bf{SSPSIR}}$ is the correction matrix from the SSP–SIR step, ${\mathbf{L}}_{i}$ is the *i*th source of the lead-field matrix, | | is norm, and ${\text{T}}$ is transpose. The obtained CC and RD values were plotted on the subject-specific cortical surfaces to illustrate to which extent signals from the different brain areas were affected by SSP–SIR.

## Results

The numbers of removed channels, removed artifact components in the SSP–SIR step and removed trials are listed in Table 1. After the cleaning, there were 3–7 clear TEP components before 200 ms in each dataset. The first component had a latency of 13–19 ms and the second 35–56 ms.

**Table 1:** The numbers of removed channels, artifact components, and trials for all the processed datasets

Subject	Stimulation site	Number of removed channels	Number of removed artifact components	Number of removed trials
S1	M1	1	4	15
S1	opIFG	9	6	35
S1	STG	13	5	48
S2	M1	11	3	25
S2	opIFG	13	3	56
S2	STG	13	4	22
S3	M1	8	6	24
S3	opIFG	12	2	33
S3	STG	12	4	10
Average		10	4	30

Here, opIFG and STG refer to frontolateral and temporoparietal areas in the right hemisphere

**Fig. 3:**
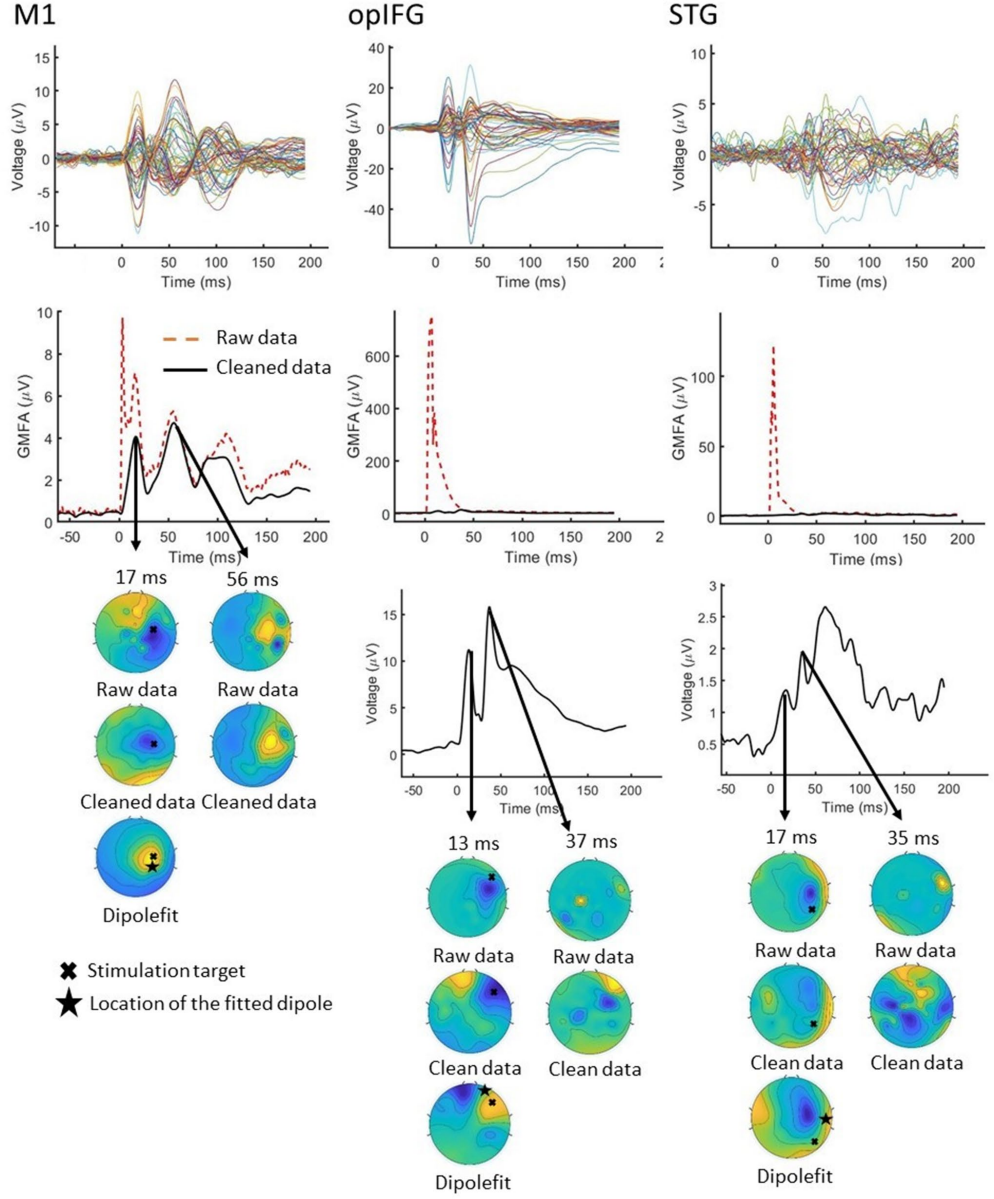
Results for processing the data with SSP–SIR after the stimulation of M1, opIFG, and STG of S1. The GMFAs of the raw and cleaned data are shown. The topographies at the latency of the first and second GMFA peaks are displayed on the right for the raw, cleaned, and calculated data from the fitted dipole. The location of the fitted dipole was checked to be suitable by comparing it to the location of the stimulation target. Then, both locations were projected to the topographic figure manually for visualization

Overall, the topography of the cleaned data and that from the fitted dipole matches well (GOF = 0.62–0.84 for M1, GOF = 0.78–0.84 for opIFG, and GOF = 0.62–0.83 for STG); the location of each fitted dipole was 2–7 cm from the stimulation site (Fig. 3).

**Fig. 4:**
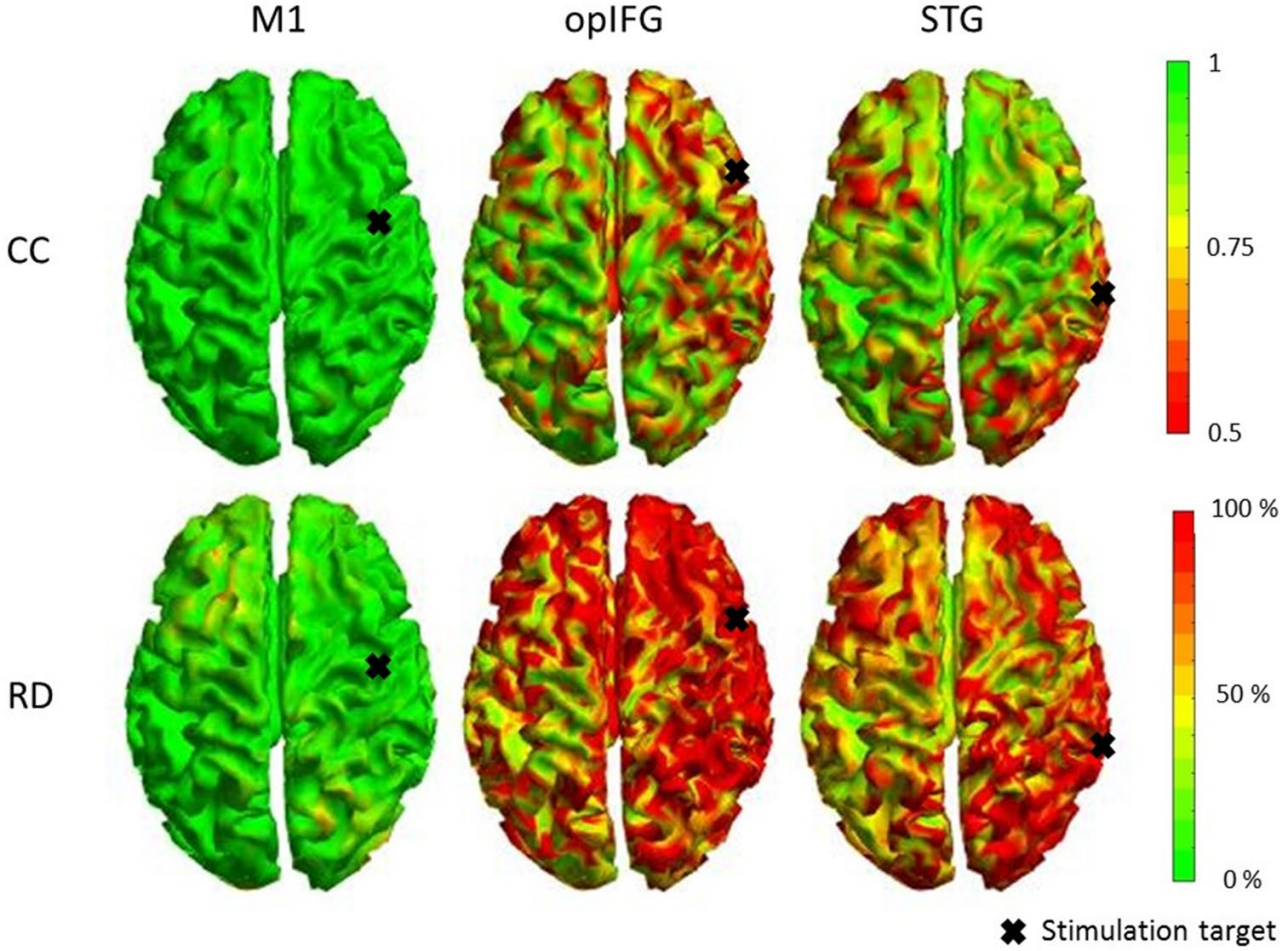
The correlation coefficients (CC) and relative differences (RD) for the neural sources on the cortex for S1. The green areas of CCs and RDs show where the data cleaning did not suppress the brain signal and the red where the data cleaning significantly suppressed the brain signal. The figures above are for the CC results for the stimulations of M1, opIFG, and STG. The figures below are the corresponding RD results

The RDs and CCs for the simulated data that were processed with the same correction matrix as the data in Fig. 3 are shown in Fig. 4. Overall, the CC values here show that the processed data correlate quite well (CC > 0.8) with the original simulated data. However, there are some areas where the correlation is not as good: for instance, the data from ipsilateral frontal areas was suppressed a lot (CC < 0.6) when opIFG was stimulated. When opIFG or STG were stimulated, the CCs were low near the stimulation site. The overall RD values for the M1 stimulation were small (RD < 20%); however, the overall RD values were high for the opIFG (RD > 80%) as well as for the STG stimulation site (RD > 50%). The RDs were highest at the stimulation site.

## Discussion

We showed that SSP–SIR is effective for removing most of the TMS-induced artifacts from EEG data when M1 or frontolateral or temporoparietal cortical areas, with large muscles, are stimulated (Mutanen et al. 2013). This was verified by visually checking whether TEP components were found after the data processing since they were not found in every dataset before the SSP–SIR step. At least three of the typical TEP components (N15, P30, N45, P55, N100, and P180) were found in each dataset. It should be emphasized that some of these responses may be at least partially of peripheral origin, e.g., elicited by the coil click. The earliest TEPs were found at 13 ms, and each dataset had at least two components with a latency of 56 ms or smaller. The topography of the first component after the cleaning and the topography of the fitted dipole were compared, showing a GOF > 0.6. The GOF values combined with the appropriate location of the fitted dipole in the stimulated hemisphere suggested that the cleaning revealed a physiologically meaningful TEP component. Although the overall GOF values did not indicate perfect fit even after cleaning, it is worth stressing that no reliable dipole fit could be done to the original data as the masking muscle artifacts prevented from identifying the early TEP components altogether.

Although brain activity components were found after artifact removal, SSP–SIR may suppress brain signals and not suppress artifacts completely. For instance, there is some residual artifact left after the SSP–SIR step when opIFG was stimulated (Fig. 3). To observe if the brain signals were suppressed after SSP–SIR, CCs and RDs were calculated. The calculated values showed that the EEG signals were mostly suppressed near the stimulation site especially when the frontolateral and temporoparietal areas were stimulated, whereas the contralateral hemisphere was nearly unaffected, allowing more straightforward source localization analysis, such as minimum-norm estimation (Hämäläinen and Ilmoniemi 1994). The calculated CCs showed also that the topographies were preserved quite well when SSP–SIR was used. Although the calculated RDs at the ipsilateral side seemed quite large, the amplitudes of the muscle artifacts are decreased remarkably by SSP–SIR. Thus, the signal-to-noise ratio at early latencies was likely considerably improved.

The use of TMS–EEG to study speech areas is limited due to large muscle artifacts (Mutanen et al. 2013; Rogasch et al. 2014), but according to the results here, SSP–SIR could be a part of the pipeline when opIFG or STG are stimulated and investigated. For instance in the future, studying, the causal connections with the presented TMS–EEG data-analysis methods could reveal more about the cognitive processing of speech as discussed in (Carreiras et al. 2012). Consequently, the investigation of individual causal connections of speech areas could help to find the optimal stimulation sites for rTMS therapies (Hamilton et al. 2011) of speech networks in, for example, stroke-related aphasia (Hamilton et al. 2011; Thiel et al. 2013; Hartwigsen et al. 2017; Heikkinen et al. 2019). The network-level mechanisms of these rTMS therapies in the rehabilitation of aphasia are still unknown but the current development in the offline data-cleaning methods shows the promise of TMS–EEG for probing these open questions. Finally, the methods presented here seem promising for probing connectivity originating from speech-related areas; however, they may not be suited for studying reactivity of these areas.

## Conclusion

SSP–SIR is an effective way to reduce the TMS-induced artifacts in EEG even when areas with large muscles inducing large artifacts are stimulated, but it also suppresses the brain signal near the stimulation site. The presented results indicate that effective connectivity of speech network may be studied with TMS–EEG, which enables individual treatment planning, for instance, when speech networks are modulated with rTMS in the rehabilitation of aphasia.
